# Supplementary material for: Exposure to violence affects the development of moral impressions and trust behavior in incarcerated males
Source: Nat Commun. 2019 Apr 26;10:1942. doi: 10.1038/s41467-019-09962-9 (PMC6486592; doi:10.1038/s41467-019-09962-9)
Supplement: Supplementary file 1 — Supplementary Information [file 41467_2019_9962_MOESM1_ESM.pdf]

Exposure to violence affects the development of moral impressions and trust behavior in incarcerated males

Supporting Information

Siegel et al.

Supplementary Methods

Simulating "good" and "bad" agents. To manipulate harm preferences, we created one agent that was more averse to harming others than the other. This was operationalized as their exchange rate between money for themselves and pain for the other individual, described with a single harm aversion parameter, κ. When $\kappa = 0$, agents are minimally harm averse and will accept any number of shocks to other individual to increase their profits; as κ approaches 1, agents become maximally harm averse and will forgo infinitely increasing amounts of money to avoid delivering a single shock. For the learning task, we created one agent who was characteristically bad ($\kappa = 0.3$), and another who was characteristically good ($\kappa = 0.7$). Effectively, this meant that the bad agent was less averse to harming the other individual and would therefore require less money to inflict pain than the good agent. Participants observed the two agents make choices for identical trial sequences. In other words, on every trial, the agents faced the same two options, but because the agents had different preferences towards harming others, they often chose differently.

Creating trial sequences. Each trial contained a pair of choices [s-, m-] and [s+, m+] that matched the indifference point of a specific κ value. We first created a set of 24 trials where values of κ were randomly drawn from a normal distribution around the good agent's indifference point (M = 0.7, s.d. = 0.15), and constrained such that $\kappa < 0.95$. Next, we created a set of 24 matched trials around the bad agent's indifference point by subtracting each κ value from 1. We wanted participants to observe identical trial sequences for the two agents, but also minimize any potential differences in learning about the agents that could be explained by discrepancies in the informational value of the trial sequence. Note that a trial with high informational value for the bad agent will have relatively low informational value for the good agent, and vice versa. Consequently, we created pairs of trials [κ, 1- κ] where the members of each pair were matched in informational value for the good and bad agent. Effectively, this meant that a trial that was highly informative about one agent's indifference point was paired with a trial that was equally informative about the other agent's indifference point. We then randomized the order of presentation of each member of the pair. The pairs comprised trials 2-49 of the sequence, while the initial and final trials were fixed to $\kappa = 0.5$.

Given a sequence of κ values, we then generated shock and money options for each κ value by generating 10,000 random pairs of positive shock movements Δs ($1 < \Delta s < 20$), and positive money movements Δm ($0.10 < \Delta m < 19.90$), and selected the pair closest to the indifference point of that κ value [Δs, Δm]. Next, these pairs were transformed into choices containing smaller amounts of shocks and money (s- and m-) and greater amounts of shocks and money (s+ and m+) as follows: s- was a positive integer between 0 and 20, randomly drawn from a uniform discrete distribution with the constraint that $0 < s- + \Delta s < 20$. Similarly, m- was a positive number between 0 and 20, randomly drawn from a uniform discrete distribution, rounded to the nearest 10th and constrained such that $0 < m- + \Delta m < 20$. s+ and m+ were then set by adding Δs and Δm to s- and m-, respectively.

We simulated the agents' decisions by computing the utility for choosing the more harmful option (V_{harm}) as a function of the agent's κ ($\kappa_{bad} = 0.3$, $\kappa_{good} = 0.7$). This model is identical to the model that best predicts human choices in the same setting[1,2].

$$V_{harm} = (1 - \kappa_n)\Delta m - \kappa_n \Delta s \tag{1}$$

Where κ_n is the κ for agent n. A softmax function was used to transform V_{harm} into a probability of choosing the more harmful option, P_{harm} :

$$P_{harm} = \frac{1}{1 + e^{-\beta \times V_{harm}}} \tag{2}$$

Where β defines the steepness of the slope in the sigmoid function. As β approaches 0 the slope become increasingly horizontal, signifying a large amount of noise in the agent's choices. As β approaches infinity the sigmoid approximates a step function, and indicates increasingly deterministic choice preferences. β was fixed to 100 to simulate agents that were completely deterministic in their choices.

$$u = [x_{rand} < P_{harm}] \tag{3}$$

Eq. (3) converts the probability of choosing the more harmful option into a binary choice, u. x_{rand} is a random number between 0 and 1.

Supplementary Notes

Supplementary Note 1

Model comparison. Three computational models were compared to describe how participants learned the agents' preferences and predicted their choices. We fit a Hierarchical Gaussian Filter model[3,4], which identified participant-specific parameters to describe each individual participant's learning process. Beliefs about an agent's harm preference were updated using a Bayesian reinforcement learning algorithm, with precision-weighted prediction errors driving belief updating at the different levels of the hierarchical model. For complete details of the HGF model as applied to this task, see Siegel and colleagues[5]. Second, we fit a Rescorla Wagner model, in which beliefs were updated by prediction errors with a fixed learning rate. Third, we fit a modified Rescorla Wagner (RW) model, in which beliefs were updated by prediction errors with separate fixed learning rates for helpful and harmful outcomes. For details about the alternative models, see **Supplementary Table 3**. The log-model evidence (LME) indicated that the HGF model (sum LME = -5920) outperforms both a simple single learning rate RW model (sum LME = -6376) and a RW model with separate learning rates for positive and negative outcomes (sum LME = -6055). We validated these findings using formal Bayesian Model Selection, which is a random-effects procedure that takes into account inter-subject heterogeneity[6,7]. To this end, we used LME data to compare between the HGF and our two RW models. This analysis yielded a protected exceedance probability indistinguishable from 1 for the HGF model for both agents, indicating effectively a 100% probability that the HGF model better explains the data than the other models included in the comparison.

Supplementary Note 2

Impression sensitivity mediates the relationship between exposure to violence and maladaptive trust. Regression analysis was used to investigate the hypothesis that subjective impression sensitivity mediates the effect of exposure to violence on adaptive trust behavior. Results indicated that ETV score was a significant predictor of subjective impression sensitivity (Δjudgment), effect = -0.025, SEM = 0.012, $p = .041$, and that subjective impression sensitivity was a significant predictor of adaptive trust behavior (Δentrust), effect = 32.582, SEM = 6.500 $p < .001$. These results support the mediational hypothesis. Exposure to violence remained a significant predictor of adaptive trust behavior after controlling for the mediator, subjective impression sensitivity, direct effect = -2.341, SEM = 0.863, $p = 0.008$. Approximately 25% of the variance in maladaptive trust behavior was accounted for by the predictors ($R^2 = .251$). The indirect effect was tested using a bootstrap estimation approach with 5000 samples. These results indicated the indirect coefficient was significant, indirect effect = -0.812, SEM = 0.414, 95% CI = -1.686, -0.045. Thus, higher exposure to violence was associated with increasingly maladaptive trust behavior as mediated by decreased subjective impression sensitivity.

It is possible that objective learning and trust are associated, such that a participant who is less able to predict the agents' choices would have greater difficulty distinguishing trust behavior for agents who behave differently. In fact, we find a significant association between accuracy and trust behavior (Spearman's *ρ, p* < 0.001), where increased accuracy was associated with a greater tendency to adapt trust behavior to agents with different harm preferences. However, there was no impact of ETV score on that relationship. Together, these findings suggest that exposure to violence does not impact the association between the ability to learn preferences of others, and moreover, use that information to engage in trust behavior. However, as demonstrated in the main analysis, exposure to violence does impact the ability to form subjective

impressions based on distinguishable behaviors, and subsequently adapt trust behavior accordingly.

Supplementary Note 3

Impression sensitivity and maladaptive trust mediates the relationship between exposure to violence and prison violations. Serial multiple mediation analysis was used to investigate the hypothesis that the extent to which one differentiates in subjective impressions and adapts trust behavior towards agents with varying harm preferences mediates the effect of exposure to violence on the number of violations in prison (**Supplementary Figure 1**). Results indicated that ETV score was a significant predictor of impression sensitivity (Δjudgment), effect = -0.025, SEM = 0.012, p = 0.041, however impression sensitivity was not an independent predictor of violations in prison, effect = 4.597, SEM = 2.735 p = 0.096. We found that ETV score also was a significant predictor of adaptive trust behavior (Δentrust), effect = -2.341, SEM = 0.863, p = .008, and that adaptive trust behavior was a significant predictor of violations in prison, effect = -0.121, SEM = 0.035 p = .001. ETV score was only a marginally significant predictor of prison violations after controlling for the mediators, direct effect = 0.622, SEM = 0.340, p = 0.070. When considering the mediating variables separately and together in relation to the mediating indirect effects of ETV score on the number of prison violations, single mediation of Δentrust was significant (indirect effect = 0.284, SEM = 0.165, 95% CI = 0.035, 0.660), and the serial-multiple mediation of Δjudgment and Δentrust was significant (indirect effect = 0.099, SEM = 0.072, 95% CI = 0.002, 0.274). The single mediation of Δjudgment was not statistically significant (indirect effect = -0.115, SEM = 0.078, 95% CI = -0.296, 0.006).

Supplementary Figures

Supplementary Figure 1. Serial Multiple Mediation Analysis.

n.s = not significant; *P < 0.05; **P < 0.01; ***P < 0.001

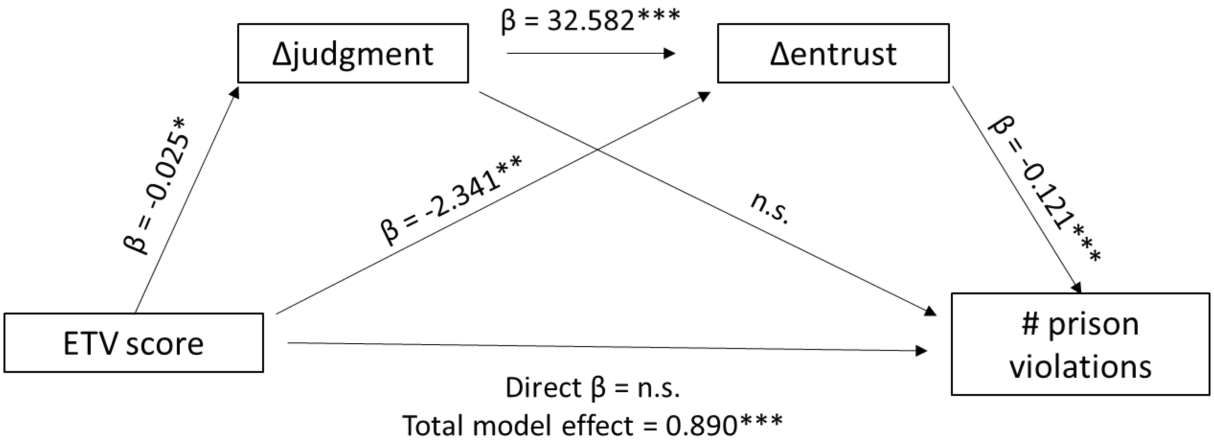

Supplementary Tables

Supplementary Table 1. Demographics

Descriptive Statistics					
	Minimum	Maximum	Mean		Std. Deviation
	statistic	statistic	statistic	Std. Error	statistic
Age on Date of Interview	20.00	58.00	34.98	0.911	9.940
Individual Income Past Year	1.00	10.00	1.61	0.165	1.796
Family Income Past Year	1.00	11.00	7.82	0.351	3.667
Highest Level of Education	5.00	16.00	11.59	0.168	1.829
PCL-R Total Score	5.30	37.0	23.37	0.649	7.080
Symptom Count Adult APD	0.00	7.00	3.82	0.163	1.775
ETV Total Score	1.00	13.00	8.04	0.295	3.219
CTQ Total Score	25.00	97.00	43.37	1.490	16.251
Total # violations in prison	0.00	93.00	7.45	1.106	12.070
Years of incarceration	0.33	30.80	6.83	0.661	7.179

Supplementary Table 2. Prior mean and variance of the perceptual and response model parameters.

Parameter	Notes		mean	variance
ω	Constant component of the tonic volatility at the second level. Represents the temporal evolution of x_2. *Estimated in native space.*		-4	1
Predictions (x_1)	Predictions are a sigmoid transformation of x_2 , and so do not have prior values.	$\mu_{1:}$	none	none
		$\sigma_{1:}$	none	none
Probabilities (x_2)	The prior mean on x_2 (prior belief about agent's harm-aversion, κ) was fixed to a neutral point that was equidistant from the true κ value of both agents. Estimated in logit space.	$\mu_{2:}$	0.5	0
	The prior variance on x_2 was fixed to ensure that any differences in learning about good and bad agents derived from the model could not result from differences in the prior estimates. Estimated in log-space.	$\sigma_{2:}$	0.35	0
β	Constant component that describes how sensitive prior beliefs are to the relative utility of different outcomes, or the prediction noise. Estimated in log-space.		1	1

Supplementary Table 3. Details of computational models.

Model	Notes	Estimated parameters
1 Learning rate Rescorla Wagner	Beliefs are symmetrically updated, with a single learning rate for each participant.	α = Learning rate<br>β = Prediction noise
2 Learning rate Rescorla Wagner	Beliefs are asymmetrically updated, with separate learning rates for positive versus negative outcomes, for each participant.	α_{pos} = Learning rate helpful outcomes<br>α_{pos} = Learning rate harmful outcomes<br>β = Prediction noise
HGF	A two-level model, with one estimated parameter governing the volatility of beliefs at the second level, and a second estimated parameter governing the prediction noise.	ω = Tonic volatility<br>β = Prediction noise

Supplementary Table 4. Results from robust regression models: Subjective impression ratings

Subjective impression ratings

	estimate	SEM	t-statistic	p-value
Intercept	0.649	0.059	11.009	<0.001
Trial	0.003	0.003	0.955	0.340
Agent	-1.300	0.075	-17.284	<0.001
ETV	-0.018	0.006	-2.902	0.004
ETV*Agent	0.037	0.009	4.222	<0.001

Subjective impression ratings controlling for age

	estimate	SEM	t-statistic	p-value
Intercept	0.658	0.078	8.459	<0.001
Trial	0.003	0.003	0.955	0.339
Agent	-1.299	0.075	-17.281	<0.001
ETV	-0.018	0.006	-2.904	0.004
Age	0.000	0.001	-0.178	0.859
ETV*Agent	0.037	0.009	4.223	<0.001

Subjective impression ratings controlling for education

	estimate	SEM	t-statistic	p-value
Intercept	0.714	0.110	6.465	<0.001
Trial	0.003	0.003	0.951	0.342
Agent	-1.300	0.075	-17.294	<0.001
ETV	-0.018	0.006	-2.963	0.003
Education	-0.005	0.008	-0.674	0.500
ETV*Agent	0.037	0.009	4.226	<0.001

Subjective impression ratings controlling for psychopathy

	estimate	SEM	t-statistic	p-value
Intercept	0.658	0.068	9.640	<0.001
Trial	0.003	0.003	0.956	0.339
Agent	-1.299	0.075	-17.272	<0.001
ETV	-0.017	0.006	-2.658	0.008
Psychopathy	-0.001	0.002	-0.256	0.798
ETV*Agent	0.037	0.009	4.217	<0.001

Subjective impression ratings controlling for APD

	estimate	SEM	t-statistic	p-value
Intercept	0.646	0.059	10.890	<0.001
Trial	0.003	0.003	0.953	0.341
Agent	-1.299	0.075	-17.275	<0.001
ETV	-0.016	0.007	-2.423	0.015
APD	-0.019	0.033	-0.578	0.563
ETV*Agent	0.037	0.009	4.213	<0.001

Subjective impression ratings controlling for CTQ

	estimate	SEM	t-statistic	p-value
Intercept	0.651	0.066	9.883	<0.001
Trial	0.003	0.003	0.955	0.340
Agent	-1.299	0.075	-17.277	<0.001
ETV	-0.018	0.006	-2.841	0.005
CTQ	0.000	0.001	-0.048	0.962
ETV*Agent	0.037	0.009	4.221	<0.001

Subjective impression ratings controlling for years of incarceration

	estimate	SEM	t-statistic	p-value
Intercept	0.643	0.059	10.885	<0.001
Trial	0.003	0.003	1.169	0.243
Agent	-1.295	0.075	-17.211	<0.001
ETV	-0.017	0.006	-2.641	0.008
Years in prison	-0.001	0.002	-0.430	0.667
ETV*Agent	0.036	0.009	4.084	<0.001

Supplementary Table 5. Results from robust regression models: Subjective uncertainty ratings

Subjective uncertainty ratings

	estimate	SEM	t-statistic	p-value
Intercept	-0.151	0.061	-2.483	0.013
Trial	-0.018	0.003	-5.969	<0.001
Agent	0.513	0.078	6.605	<0.001
ETV	0.019	0.006	2.982	0.003
ETV*Agent	-0.045	0.009	-4.973	<0.001

Subjective uncertainty ratings controlling for age

	estimate	SEM	t-statistic	p-value
Intercept	-0.194	0.080	-2.410	0.016
Trial	-0.018	0.003	-5.977	<0.001
Agent	0.514	0.078	6.611	<0.001
ETV	0.019	0.006	2.998	0.003
Age	0.001	0.001	0.822	0.411
ETV*Agent	-0.045	0.009	-4.976	<0.001

Subjective uncertainty ratings controlling for education

	estimate	SEM	t-statistic	p-value
Intercept	-0.005	0.114	-0.048	0.962
Trial	-0.017	0.003	-5.961	<0.001
Agent	0.513	0.078	6.599	<0.001
ETV	0.018	0.006	2.838	0.005
Education	-0.012	0.008	-1.506	0.132
ETV*Agent	-0.045	0.009	-4.968	<0.001

Subjective uncertainty ratings controlling for psychopathy

	estimate	SEM	t-statistic	p-value
Intercept	-0.177	0.071	-2.504	0.012
Trial	-0.018	0.003	-5.964	<0.001
Agent	0.513	0.078	6.600	<0.001
ETV	0.017	0.007	2.588	0.010
Psychopathy	0.002	0.002	0.704	0.482
ETV*Agent	-0.045	0.009	-4.969	<0.001

Subjective uncertainty ratings controlling for APD

	estimate	SEM	t-statistic	p-value
Intercept	-0.150	0.061	-2.453	0.014
Trial	-0.018	0.003	-5.972	0.000
Agent	0.513	0.078	6.601	0.000

ETV	0.019	0.007	2.687	0.007
APD	0.004	0.034	0.118	0.906
ETV*Agent	-0.045	0.009	-4.969	0.000

Subjective uncertainty ratings controlling for CTQ

	estimate	SEM	t-statistic	p-value
Intercept	-0.161	0.068	-2.370	0.018
Trial	-0.018	0.003	-5.969	<0.001
Agent	0.514	0.078	6.604	<0.001
ETV	0.019	0.006	2.866	0.004
CTQ	0.000	0.001	0.335	0.737
ETV*Agent	-0.045	0.009	-4.972	<0.001

Subjective uncertainty ratings controlling for years of incarceration on current bid

	estimate	SEM	t-statistic	p-value
Intercept	-0.152	0.061	-2.486	0.013
Trial	-0.018	0.003	-6.087	<0.001
Agent	0.517	0.078	6.632	<0.001
ETV	0.019	0.007	2.829	0.005
Years in prison	0.001	0.002	0.624	0.533
ETV*Agent	-0.045	0.009	-5.007	<0.001

Supplementary Table 6. Trust game results, including covariates

Trust

	estimate	SEM	t-statistic	p-value
Intercept	69.65048	7.096815	9.814329	<0.001
Agent	-40.1468	10.03641	-4.00012	<0.001
ETV	-1.89661	0.819505	-2.31433	0.022
ETV*Agent	3.079798	1.158956	2.657391	0.008

Trust controlling for age

	estimate	SEM	t-statistic	p-value
Intercept	61.987	9.804	6.323	<0.001
Agent	-40.171	10.033	-4.004	<0.001
ETV	-1.858	0.820	-2.267	0.024
Age	0.210	0.188	1.119	0.264
ETV*Agent	3.087	1.159	2.665	0.008

Trust controlling for education

	estimate	SEM	t-statistic	p-value
Intercept	90.93495	14.24324	6.384429	<0.001
Agent	-39.902	9.972	-4.001	<0.001
ETV	-2.037	0.817	-2.493	0.013
Education	-1.738	1.020	-1.703	0.090
ETV*Agent	3.098	1.152	2.691	0.008

Trust controlling for psychopathy

	estimate	SEM	t-statistic	p-value
Intercept	62.29476	8.487528	7.339565	<0.001
Agent	-40.348	10.087	-4.000	<0.001
ETV	-2.354	0.871	-2.702	0.007
Psychopathy	0.474	0.295	1.611	0.109
ETV*Agent	3.073	1.165	2.638	0.009

Trust controlling for APD

	estimate	SEM	t-statistic	p-value
Intercept	71.098	7.128	9.975	<0.001
Agent	-40.419	10.021	-4.034	<0.001
ETV	-2.526	0.892	-2.831	0.005
APD	7.535	4.357	1.729	0.085
ETV*Agent	3.099	1.157	2.678	0.008

Trust controlling for CTQ

	estimate	SEM	t-statistic	p-value
Intercept	60.349	8.054	7.493	<0.001
Agent	-39.775	9.983	-3.984	<0.001
ETV	-2.296	0.830	-2.767	0.006
CTQ	0.289	0.118	2.444	0.015
ETV*Agent	3.110	1.153	2.698	0.007

Trust controlling for years of incarceration

	estimate	SEM	t-statistic	p-value
Intercept	69.74759	7.168304	9.729999	<0.001
Agent	-0.007	0.278	-0.024	0.981
ETV	-39.988	10.130	-3.947	<0.001
Years in prison	-1.908	0.855	-2.233	0.027
ETV*Agent	3.070	1.174	2.614	0.010

Supplementary References

1. Crockett, M. J. *et al.* Dissociable Effects of Serotonin and Dopamine on the Valuation of Harm in Moral Decision Making. *Curr. Biol. CB* **25**, 1852–1859 (2015).
2. Crockett, M. J., Kurth-Nelson, Z., Siegel, J. Z., Dayan, P. & Dolan, R. J. Harm to others outweighs harm to self in moral decision making. *Proc. Natl. Acad. Sci.* **111**, 17320–17325 (2014).
3. Mathys, C., Daunizeau, J., Friston, K. J. & Stephan, K. E. A Bayesian foundation for individual learning under uncertainty. *Front. Hum. Neurosci.* **5**, 39 (2011).
4. Mathys, C. D. *et al.* Uncertainty in perception and the Hierarchical Gaussian Filter. *Front. Hum. Neurosci.* **8**, 825 (2014).
5. Siegel, J. Z., Mathys, C., Rutledge, R. B. & Crockett, M. J. Beliefs about bad people are volatile. *Nat. Hum. Behav.* (in press).
6. Rigoux, L., Stephan, K. E., Friston, K. J. & Daunizeau, J. Bayesian model selection for group studies - revisited. *NeuroImage* **84**, 971–985 (2014).
7. Stephan, K. E., Penny, W. D., Daunizeau, J., Moran, R. J. & Friston, K. J. Bayesian model selection for group studies. *NeuroImage* **46**, 1004–1017 (2009).
