# Supplementary material for: Exposure to violence affects the development of moral impressions and trust behavior in incarcerated males
Source: Nat Commun. 2019 Apr 26;10:1942. doi: 10.1038/s41467-019-09962-9 (PMC6486592; doi:10.1038/s41467-019-09962-9)
Supplement: Supplementary file 3 — Reporting summary [file 41467_2019_9962_MOESM3_ESM.pdf]

nature research

Corresponding author(s): Arielle Baskin-Sommers

Last updated by author(s): Mar 22, 2019

Reporting Summary

Nature Research wishes to improve the reproducibility of the work that we publish. This form provides structure for consistency and transparency in reporting. For further information on Nature Research policies, see Authors & Referees and the Editorial Policy Checklist.

Statistics

For all statistical analyses, confirm that the following items are present in the figure legend, table legend, main text, or Methods section.

n/a	Confirmed	
☐	☒	The exact sample size (*n*) for each experimental group/condition, given as a discrete number and unit of measurement
☐	☒	A statement on whether measurements were taken from distinct samples or whether the same sample was measured repeatedly
☐	☒	The statistical test(s) used AND whether they are one- or two-sided<br>*Only common tests should be described solely by name; describe more complex techniques in the Methods section.*
☐	☒	A description of all covariates tested
☐	☒	A description of any assumptions or corrections, such as tests of normality and adjustment for multiple comparisons
☐	☒	A full description of the statistical parameters including central tendency (e.g. means) or other basic estimates (e.g. regression coefficient) AND variation (e.g. standard deviation) or associated estimates of uncertainty (e.g. confidence intervals)
☐	☒	For null hypothesis testing, the test statistic (e.g. F, t, r) with confidence intervals, effect sizes, degrees of freedom and P value noted<br>*Give P values as exact values whenever suitable.*
☐	☒	For Bayesian analysis, information on the choice of priors and Markov chain Monte Carlo settings
☐	☒	For hierarchical and complex designs, identification of the appropriate level for tests and full reporting of outcomes
☐	☒	Estimates of effect sizes (e.g. Cohen's *d*, Pearson's *r*), indicating how they were calculated

Our web collection on statistics for biologists contains articles on many of the points above.

Software and code

Policy information about availability of computer code

Data collection	Data collection was conducted using Matlab (Mathworks) R2016a, and presented using the Cogent Toolbox.
Data analysis	All data analysis was completed in Matlab (Mathworks). Model parameter estimates were estimated from trial-wise predictions using the Broyden Fletcher Goldfarb Shanno optimization algorithm as implicated in the HGF toolbox, which is available at https://tnu.ethz.ch/tapas. We used the PROCESS macro for SPSS by Hayes (2012)

For manuscripts utilizing custom algorithms or software that are central to the research but not yet described in published literature, software must be made available to editors/reviewers. We strongly encourage code deposition in a community repository (e.g. GitHub). See the Nature Research guidelines for submitting code & software for further information.

Data

Policy information about availability of data

All manuscripts must include a data availability statement. This statement should provide the following information, where applicable:

- Accession codes, unique identifiers, or web links for publicly available datasets
- A list of figures that have associated raw data
- A description of any restrictions on data availability

The data that support the findings of this study are available from the corresponding author upon request.

Field-specific reporting

Please select the one below that is the best fit for your research. If you are not sure, read the appropriate sections before making your selection.

☐ Life sciences ☒ Behavioural & social sciences ☐ Ecological, evolutionary & environmental sciences

For a reference copy of the document with all sections, see nature.com/documents/nr-reporting-summary-flat.pdf

Behavioural & social sciences study design

All studies must disclose on these points even when the disclosure is negative.

Study description	Our study is a quantitative-experimental study utilizing a within-subject repeated measure design.
Research sample	The present sample included 119 male offenders (age: mean = 34.98, SEM = 0.911) from a high-security correctional institution in Connecticut. Incarcerated individuals serve as an appropriate population in which to explore how differences in exposure to violence impact harm learning. It is well-documented that exposure to violence among the incarcerated covers the full continuum of potential experiences compared to the general population where scores are often restricted in range. Moreover, by testing a sample of currently incarcerated individuals, we are better poised to investigate the variation in exposure to violence within a sample that is already demonstrating the theorized behavioral effects of such exposure.
Sampling strategy	Based on the population frame of Cheshire Correctional Institution, we employed simple random sampling.<br><br>A priori power analysis based on previous research on related topics (e.g., Siegal et al., 2018) was conducted using G*Power statistical software (Faul, Erdfelder, Lang, & Buchner, 2007). Power analyses indicated that a sample size of approximately 100 participants would result in sufficient (80%) power to detect a moderate effect finding interactions among a within-subject task variable (i.e., agent) and an individual difference measure.
Data collection	Data was collected at a high-security correctional institution in Connecticut using Matlab (Mathworks) R2016a and utilized the Cogent graphics toolbox for stimulus presentation. Responses where made using a standard computer mouse and keyboard. A researcher was present during task instructions, however no researcher was present while participants completed subsequent experimental procedures/ tasks.
Timing	4/11/16 to 7/7/17
Data exclusions	Participants were excluded from the analysis if their performance in the learning task (described in Supplementary materials section 1.1.2) was below chance (i.e., less than 50% accuracy). This criteria was pre-established and is consistent with prior research using the same paradigm (Siegel et al. ,2018)
Non-participation	None
Randomization	Participants were not allocated into experimental groups.

Reporting for specific materials, systems and methods

We require information from authors about some types of materials, experimental systems and methods used in many studies. Here, indicate whether each material, system or method listed is relevant to your study. If you are not sure if a list item applies to your research, read the appropriate section before selecting a response.

Materials & experimental systems

n/a	Involved in the study	
☒	☐	Antibodies
☒	☐	Eukaryotic cell lines
☒	☐	Palaeontology
☒	☐	Animals and other organisms
☐	☒	Human research participants
☒	☐	Clinical data

Methods

n/a	Involved in the study	
☒	☐	ChIP-seq
☒	☐	Flow cytometry
☒	☐	MRI-based neuroimaging

Human research participants

Policy information about studies involving human research participants

Population characteristics	See above.
Recruitment	A prescreen of institutional files was used to exclude individuals who had documented reading levels below the fourth-grade

Recruitment	level, who had below 70 IQ , who had diagnoses of schizophrenia, bipolar disorder, or psychosis, not otherwise specified, or who had a history of medical problems (e.g., uncorrectable auditory or visual deficits, seizures) that may have impacted their comprehension of the materials. Following the prescreen, eligible inmates were called down to the office at random and were provided with written informed consent.
Ethics oversight	Yale University Human Investigation Committee

Note that full information on the approval of the study protocol must also be provided in the manuscript.
